# New Trifluoromethyl Triazolopyrimidines as Anti-*Plasmodiumfalciparum* Agents

**DOI:** 10.3390/molecules17078285

**Published:** 2012-07-10

**Authors:** Núbia Boechat, Luiz C. S. Pinheiro, Thiago S. Silva, Anna C. C. Aguiar, Alcione S. Carvalho, Monica M. Bastos, Carolina C. P. Costa, Sergio Pinheiro, Angelo C. Pinto, Jorge S. Mendonça, Karen D. B. Dutra, Alessandra L. Valverde, Osvaldo A. Santos-Filho, Isabela P. Ceravolo, Antoniana U. Krettli

**Affiliations:** 1 Departamento de Síntese Orgânica, Instituto de Tecnologia em Fármacos-Farmanguinhos, Fundação Oswaldo Cruz, Manguinhos, Rio de Janeiro, RJ 21041-250, Brazil; 2 Departamento de Química Orgânica, Instituto de Química, Universidade Federal do Rio de Janeiro, Cidade Universitária, Rio de Janeiro, RJ 21941-590, Brazil; 3 Laboratório de Malária, Instituto de Pesquisas René Rachou, Fundação Oswaldo Cruz, Belo Horizonte, MG 30190-002, Brazil; 4 Programa de Pós Graduação em Medicina Molecular, Faculdade de Medicina, Universidade Federal de Minas Gerais, Belo Horizonte, MG 30000-000, Brazil; 5 Departamento de Química Orgânica, Instituto de Química, Universidade Federal Fluminense, Campus do Valonguinho, Niterói, RJ 24020-141, Brazil

**Keywords:** triazolopyrimidine, trifluoromethyl, malaria, *P. falciparum*, *Pf*DHODH

## Abstract

According to the World Health Organization, half of the World’s population, approximately 3.3 billion people, is at risk for developing malaria. Nearly 700,000 deaths each year are associated with the disease. Control of the disease in humans still relies on chemotherapy. Drug resistance is a limiting factor, and the search for new drugs is important. We have designed and synthesized new 2-(trifluoromethyl)[1,2,4]triazolo[1,5-*a*]pyrimidine derivatives based on bioisosteric replacement of functional groups on the anti-malarial compounds mefloquine and amodiaquine. This approach enabled us to investigate the impact of: (i) ring bioisosteric replacement; (ii) a CF_3_ group substituted at the 2-position of the [1,2,4]triazolo[1,5-*a*]pyrimidine scaffold and (iii) a range of amines as substituents at the 7-position of the of heterocyclic ring; on *in vitro* activity against *Plasmodium falciparum*. According to docking simulations, the synthesized compounds are able to interact with *P. falciparum* dihydroorotate dehydrogenase (*Pf*DHODH) through strong hydrogen bonds. The presence of a trifluoromethyl group at the 2-position of the [1,2,4]triazolo[1,5-*a*]pyrimidine ring led to increased drug activity. Thirteen compounds were found to be active, with IC_50_ values ranging from 0.023 to 20 µM in the anti-HRP2 and hypoxanthine assays. The selectivity index (SI) of the most active derivatives **5**, **8**, **11** and **16** was found to vary from 1,003 to 18,478.

## 1. Introduction

According to the World Health Organization (WHO), half of the World’s population, approximately 3.3 billion people, is at risk of contracting malaria. Nearly 700,000 deaths are associated with this disease annually. One in five childhood deaths in Africa are believed to be due to malaria [1,2].

In Brazil, a slight reduction in malaria cases was reported in 2009; however, a yearly total of over 306,000 cases was recently reported, most of which occurred in the Amazonia region [3]. Among the five known species of malaria that affect humans, three are found in Brazil: *P. falciparum*, *P. malariae* and *P. vivax*, with the latter causing 80% of the malaria cases diagnosed [3]. 

No effective vaccine is available yet for human use, although several promising antigens are undergoing clinical trials among endemic populations [4]. Control of malaria in Latin America relies on a specific therapeutic drug, chloroquine, used in association with other blood schizonticidal antimalarial drugs. Primaquine is also used in the treatment of *P. vivax* to prevent late malaria relapses caused by remaining liver forms [2]. In light of the rapid growth and spread of chloroquine-resistant *P. falciparum* and *P.vivax* strains, the development of new and more effective blood schizonticidal drugs is required. Several models are available to evaluate such new therapeutic agents [5].

The medicinal chemistry of fluorine-containing molecules has contributed greatly to the development of new drugs used in a wide range of diseases. A fluorine atom is often introduced to modify both the chemical reactivity and the physical and biological properties of organic compounds. One of the most widespread fluorine-containing functional groups in bioactive molecules is the trifluoromethyl moiety. It is a highly electronegative substituent that can exert significant electronic influence on neighboring groups. The trifluoromethyl substituent is also one of the most lipophilic groups known, making it useful for improving the targeting of molecules to enzyme active sites [6,7,8,9,10,11,12].

**Figure 1:**
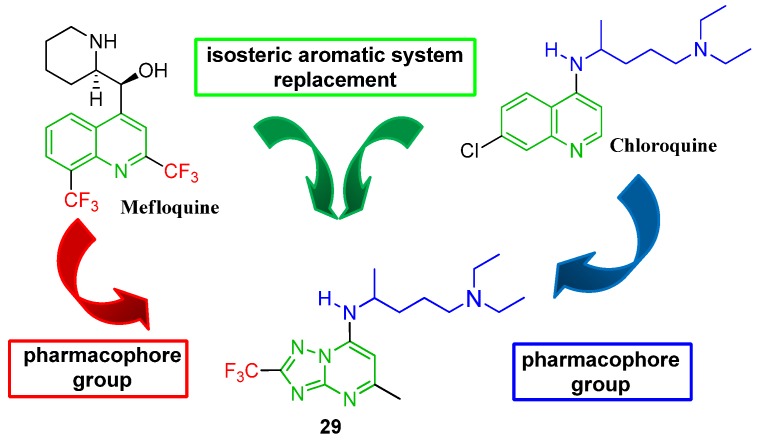
Strategy used in 2004 to obtain 5-methyl-7-*N'*-(*N,N*-diethylpentane-1,4-diamine)-2-(trifluoromethyl)[1,2,4]triazolo[1,5-*a*]pyrimidine (**29**) as an antimalarial bioisostere of quinoline.

Many heterocyclic compounds have been developed in an attempt to find new drugs to treat malaria [13,14,15,16,17,18,19,20,21,22]. In 2004, our research group described the synthesis of 5-methyl-7-*N'*-(*N*,*N*-diethylpentane-1,4-diamine)-2-(trifluoromethyl)[1,2,4]triazolo[1,5-*a*]pyrimidine derivatives [23,24]. These compounds contained a trifluoromethyl group on the [1,2,4]triazolo[1,5-*a*]pyrimidine as a ring bioisostere of mefloquine and *N^1^*,*N^1^*-diethylpentane-1,4-diamine to mimic the chloroquine pharmacophore (Figure 1). However, these compounds showed poor antimalarial activity. Phillips and co-workers recently confirmed our proposal, demonstrating that [1,2,4]triazolo[1,5-*a*]pyrimidines inhibit *P.falciparum* dihydroorotate dehydrogenase (*Pf*DHODH) and kill the parasite [25,26,27,28,29]. 

**Figure 2:**
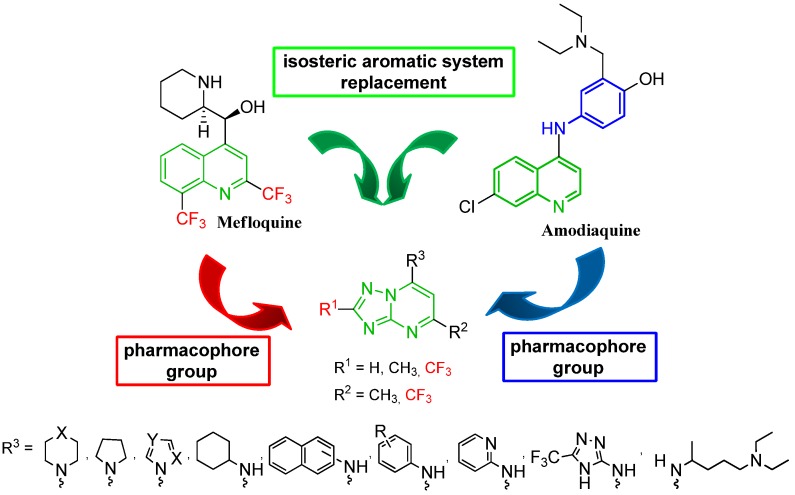
Rational approach to the design of [1,2,4]triazolo[1,5-*a*]pyrimidine derivatives 4–29.

Continuing our research by using standard medicinal chemistry and modeling principles, such as isosteric replacement, we have designed new antimalarial agents **4**–**29** that are derivatives of the [1,2,4]triazolo[1,5-*a*]pyrimidine compounds (Figure 2). These derivatives were based on ring bioisosterism with mefloquine and amodiaquine. Different arylamines **4**–**17**, **22**–**27** and aliphatic amines **18**–**21**, **28**, **29** were incorporated into the structure to investigate the importance of the substituent at the 7-position. Investigation of the impact of CF_3_ in the 2-position of the [1,2,4]triazolo[1,5-*a*]pyrimidine scaffold was prioritized. Twenty-six derivatives **4**–**29** from this series were designed, synthesized and evaluated *in vitro* against a *Plasmodium falciparum* chloroquine-resistant W2 clone strain.

## 2. Results and Discussion

### 2.1. Synthesis of Compounds

**Figure 3:**
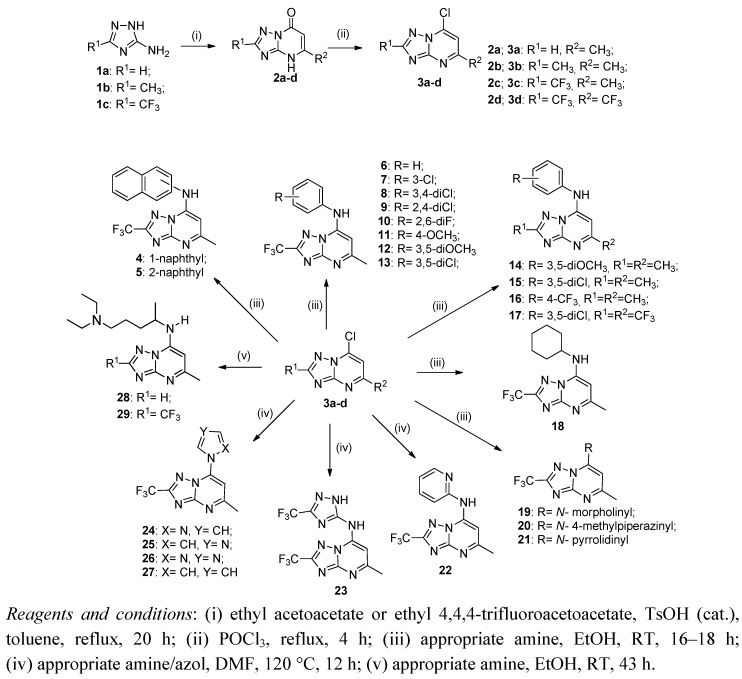
Synthesis of [1,2,4]triazolo[1,5-*a*]pyrimidines 4–29.

The synthetic route to [1,2,4]triazolo[1,5-*a*]pyrimidine derivatives **4**–**29** is shown in Figure 3. Condensation of 3-amino-1,2,4-triazoles **1a**–**c** with ethyl acetoacetate or ethyl 4,4,4-trifluoroacetoacetate in refluxing toluene, in the presence of catalytic *p*-toluenesulfonic acid, gave [1,2,4]triazolo[1,5-*a*]pyrimidin-7(4*H*)-ones **2a**–**d** in 50–90% yields after 24 h, as previously described in the literature [23,24,30,31]. Spectroscopic data of compounds **2a**–**d** were in agreement with the literature, and X-ray crystallography showed **2c** as their keto-tautomers [24]. Compounds **2a**–**d** were easily chlorinated with phosphorus oxychloride under reflux for 6 h, affording the respective 7-chloro[1,2,4]triazolo[1,5-*a*]pyrimidines **3a**–**d** in 58–90% yields. Compounds **3a** and **3b** showed identical spectroscopic data to those reported in the literature [23,25,26,27,28,29,30,31]. Spectroscopic data of compounds **3c** and **3d** were in agreement with the proposed structures. Reaction of compounds **3a**–**d** with several amines produced the target compounds **4**–**29** in 30–90% yields.

### 2.2. Molecular Modeling

It is known that [1,2,4]triazolo[1,5-*a*]pyrimidine derivatives interact with the enzyme dihydroorotate dehydrogenase (DHODH) [25,32]. Thus, docking calculations were performed for the newly synthesized compounds to verify their binding modes with this enzyme from*P. falciparum* (*Pf*DHODH) available in the Protein Data Bank as PDB ID code 3I65. Each compound was modeled, and 1,000 steps of energy minimization were performed by the steepest descent method using Gasteiger-Hückel charges and a dielectric constant of 80 in the Tripos force field [33]. The structures were further optimized using the conjugated gradient method. 

Ligand-enzyme docking simulations were performed with the molecular docking algorithm MolDock [34] using the Molegro Virtual Docker 4.3.0. MolDock uses a heuristic search algorithm (*i.e*., termed guided differential evolution), which combines differential evolution and a cavity-prediction algorithm. The docking scoring function is an extension of the piecewise linear potential (PLP) [34]. After the ligands and protein coordinates were imported, all structural parameters, including bond type, hybridization, explicit hydrogen, charges, and flexible torsions, were assigned using the automatic preparation function in the Molegro Virtual Docker software. For each compound, 100 docking runs were performed with the initial population of 150 individuals. After each compound was docked, it was energy-minimized into the active site of the enzyme.

All synthesized compounds were docked into *Pf*DHODH. The known *Pf*DHODH inhibitor DSM1 that was co-crystallized with the enzyme was used as the reference molecule during the docking simulations [32].

**Figure 4:**
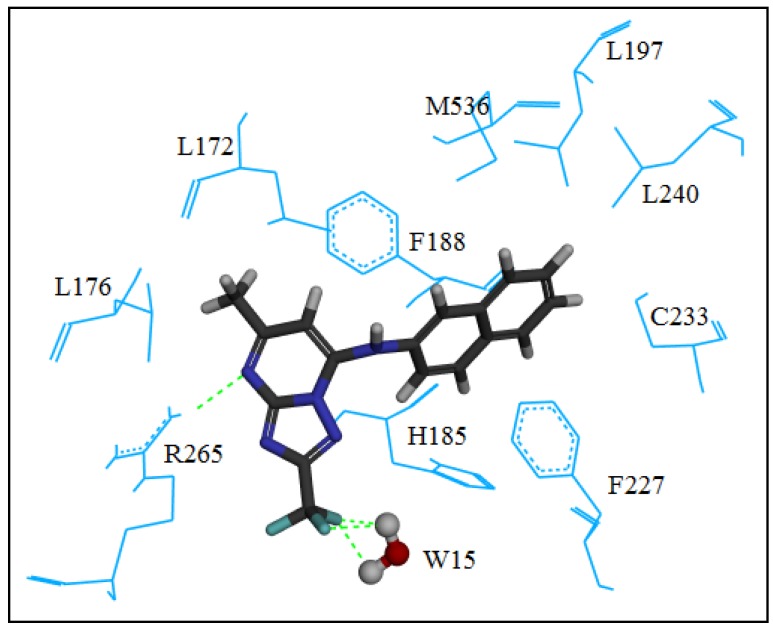
Compound **5** docked into the active site of *Pf*DHODH.

Enzyme residues H185 and R265 and the water molecule W15 found in the crystal structure of *Pf*DHODH act as “molecular anchors” for binding molecules **4**–**29** at the active site. Such “anchors” are actually hydrogen bonds formed between **4**–**29** and the enzyme residues or the water molecule. Each [1,2,4]triazolo[1,5-*a*]pyrimidine **4**–**29** interacts with R265 by forming a hydrogen bond through N-4. An additional hydrogen bond can be present between N-1 of the pyrimidine ring and H185. The frequency with which hydrogen bonds are formed between the W15 molecule and CF_3_ groups at the 2-position of [1,2,4]triazolo[1,5-*a*]pyrimidine rings of the majority of compounds was remarkable. Twenty-two of the 26 synthesized compounds (compounds **4**–**13**, **17**–**27**, **29**) showed the CF_3_–W15 interaction. Consequently, the CF_3_ group must be carefully considered for the development of potential new lead inhibitors of *Pf*DHODH. Figure 4 shows the R265–N-4 and the CF_3_–W15 interactions.

### 2.3. Continuous Cultures and *in Vitro* Assays with P. falciparum-Infected Erythrocytes

The *P. falciparum* W2 clone, which is chloroquine-resistant and mefloquine-sensitive [35], was maintained in continuous culture. Briefly, the parasites were kept as described [36] at 37 °C in human erythrocytes (A^+^) in complete medium (RPMI 1640 supplemented with 10% human sera blood group A^+^, 2% glutamine, and 7.5% NaHCO_3_) either in Petri dishes in a candle jar or in 25-cm culture flasks in an environment containing a gas mixture atmosphere (3% O_2_, 5% CO_2_ and 91% N_2_). Before testing, the ring-stage parasites were synchronized by sorbitol [37]; the suspension was adjusted for parasitemia and hematocrit as described below for each test used. The infected red blood cells were distributed in a 96-well microtiter plate (Corning, Santa Clara, CA, USA), 180 μL/well, to which 20 μL of different concentrations of test drugs and controls had previously been added. The maximum concentration 50 µg/mL (~157 μM) was tested two or three times; compounds are considered inactive at equal or higher doses.

The effects of compounds against the W2 *P. falciparum* blood cultures were evaluated through incorporation of ^3^H hypoxanthine (Perkin Elmer, Waltham, MA, USA) by the parasites [38]. Alternatively, compound effects were also examined using monoclonal antibodies to a commercially available parasite histidine and alanine-rich protein (HRP2) (MPFM ICLLAB-55A^®^, MPFG55P ICLLAB^®^, Immunology Consultants Laboratory Inc. (ICL), Portland, OR, USA), as described previously [39]. The [3H]-hypoxanthine assay was performed with 1% of parasitemia and 1% of hematocrit, and the level of isotope incorporation was read in a beta-counter (Perkin Elmer). The anti-HRP2 test was performed with 0.05% parasitemia and 1.5% hematocrit, and the quantification of protein was determined using a specific read at 450 nm in a spectrophotometer (SpectraMax340PC^384^, Molecular Devices, Sunnyvale, CA, USA). Drug activities were expressed by the half-maximal inhibitory dose (IC_50_) compared to the drug-free controls and estimated using the curve-fitting software Origin 8.0 (OriginLab Corporation, Northampton, MA, USA) [40].

### 2.4. Cell Cultures and Cytotoxicity Tests

The human hepatoma cell line (HepG2) was cultured in 75-cm^2^ sterile flasks with RPMI 1640 medium supplemented with 10% heat-inactivated fetal and 40 mg/L gentamicin in a 5% CO_2_ atmosphere at 37 °C. For *in vitro* cytotoxicity experiments, the cell monolayer was trypsinized, washed with culture medium, distributed in a flat-bottomed 96-well plate (5 × 10^3^ cells/well), and finally incubated for 18 h at 37 °C for cell adherence. 

For cytotoxicity testing, we used the (3-(4,5-dimethylthiazol-2-yl)-2,5-diphenyltetrazolium bromide (MTT) assay, performed as described in the literature [41]. The HepG2 cell line was incubated with 20 µL of the compounds in different concentrations (200–25 µg/mL) for 24 h in an atmosphere of 5% CO_2_ at 37 °C. 

For the MTT assay, which evaluates mitochondrial viability, 20 µL of MTT solution (5 mg/mL) was added, and the plates were incubated for an additional 3 h. After incubation, the supernatant was carefully removed from the wells, followed by addition of 100 µL DMSO with thorough mixing. Optical densities at 570 and 630 nm (background) were determined by an ELISA reader.

Cell viability was expressed as the percentage of control absorbance obtained in untreated cells after subtracting the absorbance from appropriate background. Lastly, the minimum lethal dose for 50% of the cells (MLD_50_) was determined as previously described in the literature [42]. The ratio between MLD_50_ and drug activity (IC_50_) *in vitro* was used to determine the selectivity index (SI).

The synthesized compounds with different substituents in the 2-, 5-, and 7-positions of the [1,2,4]triazolo[1,5-*a*]pyrimidine scaffold were tested against the W2-chloroquine-resistant *P. falciparum* clone. The anti-*P. falciparum* activity and cytotoxicity results of the 26 new [1,2,4]triazolo[1,5-*a*]pyrimidine derivatives are summarized in Table 1. Among them, 13 showed low IC_50_ values (ranging from 0.023 to 20 µM) in the anti-HPR2 and [3^H^]-hypoxanthine incorporation tests. The other compounds showed high IC_50_ values (>20 µM) or were inactive. Values of SI greater than 10 are considered non-toxic, which was the case for all new compound tested herein. Derivatives **5**, **8**, **11** and **16** were the most active, least toxic, and had the highest SI values (from 1,003 to 18,478). 

**Table 1:** Evaluation of anti-plasmodial activity against a chloroquine-resistant W2 clone of *P. falciparum*, cytotoxicity against a human hepatoma cell line (HepG2) and drug selectivity index (SI) of compounds 4–29 and chloroquine.

Compounds	IC_50_ (µM) * *Plasmodium Falciparum*		MDL_50_ HepG2 (µM)	SI MDL_50_/IC_50_
	Anti-HRP2	Hypoxanthine		
**4**	2.2	Nd	326	148
**5**	0.023 ± 0.002	Nd	425	18,478
**6**	3 ± 2	10.2 ± 5	>682	>227
**7**	1.83 ± 1.10	1.22	373	203
**8**	0.55 ± 0.66	0.11 ± 0.05	>552	1,003
**9**	2.7 ± 2.21	3.32 ± 1.10	320	118
**10**	>69.9	>148.9	337	Inactive
**11**	0.4 ± 0.09	1.5 ± 1.2	>619	>1,547
**12**	2.5 ± 0.05	0.36 ± 0.1	498	>199
**13**	1.47 ± 0.11	0.83 ± 0.58	320	218
**14**	3.84 ± 0.40	15.05 ± 10.03	515	134
**15**	>162.3	149.3	415	Inactive
**16**	0.3	0.4 ± 0.06	446	1486
**17**	12.31	23.42	269	Inactive
**18**	8.69 ± 0.46	10.03	515	64
**19**	>174.0	>174.0	>697	Inactive
**20**	39 ± 17	57 ± 13	>666	Inactive
**21**	>184.5	>184.5	>738	Inactive
**22**	>156.4	>170.0	394	Inactive
**23**	36.93 ± 11.36	31.25 ± 17.04	<71	Inactive
**24**	2.1	Nd	>746	>355
**25**	21 ± 2	15 ± 0.7	<93	Inactive
**26**	29.73	33.45 ± 14.86	<93	Inactive
**27**	97.37 ± 22.47	78.65 ± 7.49	>749	Inactive
**28**	>172.4	>172.4	>689	Inactive
**29**	73 ± 17	39.1	>558	Inactive
**Chloroquine**	0.22	0.23	490	4,200

* IC_50_ < 10 µM active; >10 and <20 µM partially active; >20 µM inactive; Nd: not determined.

Taken together, the *in vitro* data of anti-*P. falciparum* and its toxicological activities show that these four compounds are promising candidates for the development of a novel anti-malarial therapy. Decreased biological activity resulted from substitution with cyclic secondary amines **19**–**21** and alkylamines **28**, **29**. Moreover, except in the case of pyrazolyl derivative **24**, compounds containing azoles or heteroaromatics as substituents were inactive (compounds **22**, **23**, **25**–**27**).

## 3. Experimental

^1^H-, ^13^C- and ^19^F-Nuclear Magnetic Resonance (NMR) spectra were obtained at 400.00 MHz, 100.00 MHz and 376.00 MHz, respectively, on a Bruker Avance instrument equipped with a 5-mm probe, using tetramethylsilane as the internal standard. Chemical shifts (δ) reported in ppm and coupling constants (*J*) in Hertz. Fourier transform infrared (FT-IR) absorption spectra were recorded on a Shimadzu mode IR Prestige-21 spectrophotometer by reflectance in KBr. GC/MS experiments were conducted using a model 6,890 N gas chromatograph (Agilent, Palo Alto, CA, USA) equipped with a 7,683 B auto sampler coupled with a model MS 5,973 N single quadrupole mass spectrometer (Agilent). The GC was equipped with a HP-5MS capillary column 30 m in length, 0.25 mm in diameter, with a 0.25-μm film thickness. The temperature program began at 50 °C, then increased to 300 °C at a rate of 10 °C/min and held for 10 minutes. The helium flow rate was 0.5 mL/min. Melting points (m.p.) were determined with a Büchi model B-545 apparatus. TLC was carried out using silica gel F-254 glass plates (20 × 20 cm). All other reagents and solvents used were analytical grade.

### 3.1. General Procedure for Preparing [1,2,4]Triazolo[1,5-a]pyrimidin-7(4H)-ones *(2a–d)*

The [1,2,4]triazolo[1,5-*a*]pyrimidin-7(4*H*)-ones **2a**,**b** were prepared according to methodology described in the literature [23,24,30,31]. A mixture of a 3-amino-1,2,4-triazole derivative (**1c**) (20 mmol) and ethyl acetoacetate or ethyl 4,4,4-trifluoroacetoacetate (15 mL) was stirred at room temperature for 30 minutes. To the mixture was added toluene (30 mL) and catalytic *p*-toluenesulfonic acid. The reaction was heated under reflux for 24 h. The resulting solid was then cooled to RT, filtered, washed with toluene, and dried. Compounds **2c**,**d** were used without purification.

*5-Methyl-2-*(trifluoromethyl)[*1,2,4*]*triazolo*[*1,5-a*]*pyrimidin-7*(*4H*)*-one* (**2c**) Yield: 90%; m.p. 266–267 °C; IR (KBr, cm^−1^): 3,445 (NH); 1702 (C=O); 1,659 (C=N); 761 (CF_3_). ^1^H-NMR (CD_3_OD, δ): 2.34 (s, 3H, CH_3_); 5.98 (s, 1H, H-6); 13.59 (s, 1H, NH). ^13^C-NMR (CD_3_OD, δ): 18.75; 99.26; 119.20 (q, ^1^*J* = 268.8 Hz, C-9); 151.56; 151.99 (q, *J* = 39 Hz, C-2); 152.74; 155.27. ^19^F-NMR (CD_3_OD, δ): −64.97 (s, 3F). GC-MS (70 eV) *m/z* (%): 218; 199; 190; 69 (100); 68; 53.

*2*,*5-bis(Trifluoromethyl*)[*1,2,4*]*triazolo*[1,5-*a*]*pyrimidin-7*(*4H*)*-one* (**2d**) Yield: 50%; m.p. 176–178 °C; IR (KBr, cm^−1^): 3,489 (NH); 1,708 (C=O); 1,608; 761 (CF_3_). ^1^H-NMR (CD_3_OD, δ): 6.61 (s, 1H, H-6). ^13^C-NMR (CD_3_OD, δ): 101.33; 120.50 (q, ^1^*J* = 268.0 Hz); 121.27 (q, ^1^*J* = 272.0 Hz); 142.92 (q, *J* = 37.5 Hz); 153.53. ^19^F-NMR (CD_3_OD, δ): −67.73 (s, 3F); −67.73 (s, 3F). GC-MS (70 eV) *m/z* (%): 272; 152 (100); 69; 53; 43.

### 3.2. General Procedure for Preparing 7-Chloro[1,2,4]triazolo[1,5-*a*]pyrimidines *3a–d*

The 7-chloro[1,2,4]triazolo[1,5-*a*]pyrimidine **3a**,**b** were prepared according to methodology described in the literature [23,30,31]. To a [1,2,4]triazolo[1,5-*a*]pyrimidin-7(4*H*)-one derivative **2c**,**d** (4.5 mmol) was added phosphorus oxychloride (10 mL). The mixture was stirred under reflux for 4 h. Excess solvent was removed under reduced pressure and the resulting material was carefully added to 50 mL of crushed ice, which was then basified to pH 12 with NaOH (6 M aq.) and stirred for 40. The mixture was diluted with water (30 mL) and extracted with chloroform (3 × 30 mL). The combined organic solution was washed with water (3 × 50 mL), dried (magnesium sulfate), filtered, and concentrated under vacuum.

*7-Chloro-5-methyl-2-*(*trifluoromethyl*)[*1,2,4*]*triazolo*[*1,5-a*]*pyrimidine* (**3c**). Yield: 90%; m.p. 98–100 °C. IR (KBr, cm^−1^): 3,062 (CH); 1,614 (C=N); 1,523; 1,487; 1,062; 1,215; 1,190; 752 (CF_3_). ^1^H-NMR (CD_3_OD, δ): 2.74 (s, 3H, CH_3_); 7.65 (s, 1H, H-6). ^13^C-NMR (CD_3_OD, δ): 25.31; 115.18; 120.74 (q, *J* = 268.6 Hz, C-9); 157.52 (q, *J* = 41.6 Hz, C-5); 141.32; 170.14. ^19^F-NMR (CD_3_OD, δ): −67.27 (s, 3F). GC-MS (70 eV) *m/z* (%): 238; 236; 216; 201; 76; 69 (100); 51.

*7-Chloro-2,5-bis*(*trifluoromethyl*)[*1,2,4*]*triazolo*[*1,5-a*]*pyrimidine* (**3d**). Yield: 80%; m.p. 107–108 °C. IR (KBr, cm^−1^): 3,130 (CH); 1,639 (C=N); 823; 625; 684 (CF_3_). ^1^H-NMR (CD_3_OD, δ): 7.68 (s, 1H, H-6). ^13^C-NMR (CD_3_OD, δ): 112.39; 117.90 (q, *J* = 274.0 Hz); 118.60 (q, *J* = 270.0 Hz); 137.30 (q, *J* = 40.4 Hz); 155.29; 158.37; 158.90 (q, *J* = 40.7 Hz). ^19^F-NMR (CD_3_OD, δ): −68.22 (s, 3F); −65.83 (s, 3F). GC-MS (70 eV) *m/z* (%): 292; 290; 271; 69 (100).

### 3.3. General Procedure for Preparing 5-Methyl-7-aryl/cycloalkylamine[1,2,4]triazolo[1,5-a]pyrimidines *4–21*

A mixture of a 7-chloro[1,2,4]triazolo[1,5-*a*]pyrimidine derivative **3b**–**d** and the appropriate amine (1 equivalent) in ethanol (10 mL) was stirred at room temperature for 16–18 h. The reaction mixture was concentrated and poured into 50 mL of ice-cold water. The precipitate was collected by filtration and washed with water (10 mL) to give **4**–**21** in yields of 50–90%.

*5-Methyl-7-*(*naphthalen-1-ylamine*)*-2-*(*trifluoromethyl*)[*1,2,4*]*triazolo*[*1,5-a*]*pyrimidine* (**4**). Yield: 85%; m.p. 186–188 °C. IR (KBr, cm^−1^): 3,516 (N-H); 3,304; 1,627 (C=N); 1,575; 1,514; 1,448; 1,396; 1,369; 1,301; 1,282; 1,211, 1,195; 1,139; 806; 781 (CF_3_). ^1^H-NMR (CD_3_OD, δ): 2.36 (s, 3H, CH_3_); 5.90 (s, 1H, H-6); 7.65–7.55 (m, 4H, H-5'-H-8'); 8.01–7.95 (m, 3H, H-2'-H-4'). ^13^C-NMR (CD_3_OD, δ): 25.03; 92.49; 121.13 (q, *J* = 268.6 Hz, C-9); 123.54; 126.64; 127.09; 128.14; 128.51; 129.83; 130.25; 131.43; 133.17; 136.40; 150.15; 156.98 (d; *J* = 39.3 Hz; C-2);157.42; 168.41. ^19^F-NMR (CD_3_OD, δ): −67.21 (s, 3F). GC/MS *m/z* (%): 343 (100); 324; 274; 190; 177; 127. 

*5-Methyl-7-*(*naphthalen-2-ylamine*)*-2-*(*trifluoromethyl*)[*1,2,4*]*triazolo*[*1,5-a*]*pyrimidine* (**5**). Yield: 90%; m.p. 163–164 °C. IR (KBr, cm^−1^): 3,062 (NH); 1,662 (C=N); 1,589; 1,506; 1,467; 1,369; 1,309; 1,288; 1,192; 1,184; 1,153; 848; 829; 779 (CF_3_). ^1^H-NMR (CD_3_OD, δ): 2.60 (s, 3H, CH_3_); 6.67 (s, 1H, H-6); 7.63–7.58 (m, 3H, H-1'-H-4'); 7.97–8.11 (m, 4H, H-5'-H-8'). ^13^C-NMR (CD_3_OD, δ): 25.19; 92.31; 121.07 (q, *J* = 214.8 Hz, C-9); 123.94; 124.41; 127.63; 128.14; 128.85; 128.95; 131.02; 133.62; 134.91; 135.31; 148.63; 156.71 (q, *J* = 31.3 Hz, C-2); 157.32; 168.57. ^19^F-NMR (CD_3_OD, δ): −67.47 (s, 3F). GC/MS *m/z* (%): 343 (100); 328; 247; 190; 177; 127.

*5-Methyl-7-phenylamine-2-*(*trifluoromethyl*)[*1,2,4*]*triazolo*[*1,5-a*]*pyrimidine* (**6**). Yield: 85%; m.p. 151–152 °C. IR (KBr, cm^−1^): 3,653 (N-H); 3,062 (CH-aromatic); 2,999; 1,631 (C=N); 1,589; 1,512; 1,429; 1,369; 1,305; 1,286; 1,197, 1,170; 1,139; 777; 721 (CF_3_). ^1^H-NMR (CD_3_OD, δ): 2.48 (s, 3H, CH_3_); 6.46 (s, 1H, H-6); 7.50–7.36 (m, 5H, H-2'-H-6'). ^13^C-NMR (CD_3_OD, δ): 25.18; 92.13; 121.05 (q, *J* = 268.5 Hz, C-9); 126.28; 128.49; 131.08; 137.56; 148.83; 156.79 (q, *J* = 39 Hz, C-2); 157.39; 168.55. ^19^F-NMR (CD_3_OD, δ): −67.22 (s, 3F). GC/MS *m/z* (%): 293 (100); 294; 278; 224; 190; 177; 77. 

*7-*(*3-Chlorophenylamine*)*-5-methyl-2-*(*trifluoromethyl*)[*1,2,4*]*triazolo*[*1,5-a*]*pyrimidine* (**7**). Yield: 50%; m.p. 136–138 °C. IR (KBr, cm^−1^): 3,603 (NH); 3,062 (CH-aromatic); 2,893; 1,627 (C=N); 1,589; 1,504; 1,431; 1,323; 1,192; 1,141; 1,072; 848; 779 (CF_3_). ^1^H-NMR (CD_3_OD; δ): 2.52 (s, 3H, CH_3_); 6.55 (s, 1H, H-6); 7.38 (d, *J* = 7.5 Hz, H-6'); 7.42 (d, *J* = 8 Hz, 1H, H-4'); 7.50 (t, *J* = 8.0 Hz, 1H, H-'); 7.51 (d, *J* = 2.0 Hz, 1H, H-2'). ^13^C-NMR (CD_3_OD, δ): 25.23; 92.46; 121.02 (q, *J* = 214.9 Hz, C-9); 124.42; 126.17; 128.34; 132.31; 136.49; 139.15; 148.43; 156.77 (q, *J* = 31.3 Hz, C-2); 157.34; 168.85. ^19^F-NMR (CD_3_OD, δ): −67.20 (s, 3F). GC/MS *m/z* (%): 327 (100); 328; 329; 312; 258; 190; 177; 111.

*7-*(*3,4-Dichlorophenylamine*)*-5-methyl-2-*(*trifluoromethyl*)[*1,2,4*]*triazolo*[*1,5-a*]*pyrimidine* (**8**). Yield: 90%; m.p. 136–138 °C. IR (KBr, cm^−1^): 3,606 (NH); 3,062; 3,005 (CH-aromatic); 2,360; 1,672 (C=N); 1,585; 1,512; 1,469; 1,388; 1,315; 1,288; 1,195; 1,149; 833; 779 (CF_3_).^1^H-NMR (CD_3_OD, δ): 2.53 (s, 3H, CH_3_); 6.57 (s, 1H, H-6); 7.43 (dd, *J* = 2.0 Hz; *J* = 8.75 Hz, 1H, H-6'); 7.65 (d, *J* = 9.0 Hz, 1H, H-5'); 7.66 (d, *J* = 2.5 Hz, 1H, H-2'). ^13^C-NMR (CD_3_OD, δ): 25.23; 92.66; 121.03 (q, *J* = 268.5 Hz, C-9); 125.85; 128.05; 131.77; 132.79; 134.61; 137.78; 148.31; 156.86 (q, *J* = 39.2 Hz, C-2); 157.38; 168.97. ^19^F-NMR (CD_3_OD, δ): −67.27 (s; 3F). GC/MS *m/z* (%): 361 (100); 362; 363; 346; 292; 190; 177; 145.

*7-*(*2,4-Dichlorophenylamine*)*-5-methyl-2-*(*trifluoromethyl*)[*1,2,4*]*triazolo*[*1,5-a*]*pyrimidine* (**9**). Yield: 80%; m.p. 217–218 °C. IR (KBr, cm^−1^): 3,128 (NH); 3,066 (CH-aromatic); 1,624 (C=N); 1,577; 1,508; 1,469; 1,307; 1,288; 1,188; 1,168; 1,141; 1,099; 1,056; 844; 779 (CF_3_). ^1^H-NMR (CD_3_OD, δ): 2.58 (s, 3H, CH_3_); 6.20 (s, 1H, H-6); 7.60 (dd, *J* = 2.0 Hz, *J* = 6.8 Hz, 1H, H-5'); 7.65 (d, *J* = 6.8 Hz, 1H, H-6'); 7.82 (d, *J* = 2.0 Hz, 1H, H-3'). ^13^C-NMR (CD_3_OD, δ): 25.13; 92.78; 121.02 (q, *J* = 215 Hz, C-9); 130.08; 131.74; 131.81; 133.52; 134.42; 135.94; 148.69; 156.96 (q, *J* = 31 Hz, C-2); 157.29; 168.85. ^19^F-NMR (CD_3_OD, δ): −67.17 (s, 3F). GC/MS *m/z* (%): 361; 346; 328; 326 (100); 190; 177; 145.

*7-*(*2,6-Difluorophenylamine*)*-5-methyl-2-*(*trifluoromethyl*)[*1,2,4*]*triazolo*[*1,5-a*]*pyrimidine* (**10**). Yield: 80%; m.p. 129–130 °C. IR (KBr, cm^−1^): 3,383 (NH); 3,082; 2,357; 1,631 (C=N); 1,577; 1,516; 1,477; 1,373; 1,296; 1,242; 1,195; 1,161; 1,006; 794 (CF_3_). ^1^H-NMR (CD_3_OD, δ): 2.51 (s, 3H, CH_3_); 6.14 (s, 1H, H-6); 7.22 (m, 2H, H-3', H-5'); 7.55–7.49 (m, 1H, H-4'). ^13^C-NMR (CD_3_OD, δ): 25.12; 92.81; 113.82 (dd; *J* = 15.45 Hz, *J* = 3.2 Hz, 2C, C-3', C-5'); 121.01 (q, *J* = 214.9 Hz, C-9); 131.49 (t;*J* = 7.15 Hz; 2C; C-1', C-4'); 148.69; 157.02 (q, *J* = 31.5 Hz; C-2); 157.25; 160.36 (dd; *J* = 200.2 Hz, *J* = 2.2 Hz, 2C, C-2', C-6'); 169.01. ^19^F-NMR (CD_3_OD, δ): −67.29 (s; 3F); −119.77 (s; 2F). GC/MS *m/z* (%): 329 (100); 330; 310; 260; 190; 177.

*7-*(*4-Methoxyphenylamine*)*-5-methyl-2-*(*trifluoromethyl*)[*1,2,4*]*triazolo*[*1,5-a*]*pyrimidine* (**11**). Yield: 60%; m.p. 163–164 °C. IR (KBr, cm^−1^): 3,606 (NH); 3,007 (CH-aromatic); 2,050; 1,629 (C=N); 1,606; 1,579; 1,510; 1,433; 1,377; 1,288; 1,269; 1,242; 1,031; 1,193, 1,168; 1,136, 823; 779 (CF_3_). ^1^H-NMR (CD_3_OD, δ): 2.46 (s, 3H, CH_3_); 3.84 (s, 3H, OCH_3_); 6.29 (s. 1H, H-6); 7.03 (d; *J* = 9.2 Hz, 2H, H-2'; H-6'); 7.34 (d, *J* = 9.2 Hz, 2H, H-3', H-5'). ^13^C-NMR (CD_3_OD, δ): 25.11; 56.22; 91.88; 116.28; 121.04 (q, *J* = 268.9 Hz; C-9); 128.27; 129.88; 149.45; 156.79 (d, *J* = 38.7 Hz, C-2); 157.38; 160.62; 168.36. ^19^F-NMR (CD_3_OD, δ): −67.24 (s, 3F). GC/MS *m/z* (%): 323 (100); 324; 308; 254; 190; 177.

*7-*(*3,5-Dimethoxyphenylamine*)*-5-methyl-2-*(*trifluoromethyl*)[*1,2,4*]*triazolo*[*1,5-a*]*pyrimidine* (**12**). Yield: 90%; m.p. 154–156 °C. IR (KBr, cm^−1^): 3,383 (NH); 3,007 (CH-aromatic); 2,968; 2,843; 1,629 (C=N); 1,604; 1,581; 1,502; 1,431; 1,346; 1,309; 1,284; 1,251; 1,205; 1,155; 1,053; 1,155; 979; 839; 777 (CF_3_). ^1^H-NMR (CD_3_OD, δ in ppm): 2.50 (s, 3H, CH_3_); 3.79 (s, 6H, OCH_3_); 6.55 (s, 1H, H-6); 6.44 (t, *J* = 2.4 Hz, 1H, H-4'); 6.58 (d, *J* = 2.4 Hz; 2H; H-2', H-6’). ^13^C-NMR (CD_3_OD, δ in ppm): 25.08; 56.20; 92.68; 100.08; 104.29; 121.01 (q, *J* = 269 Hz, C-9); 139.06; 148.67; 156.75 (q, *J* = 39 Hz, C-2); 157.19; 163.38; 168.36. ^19^F-NMR (CD_3_OD, δ in ppm): −67.19 (s, 3F). GC/MS *m/z* (%): 353 (100); 354; 352; 338; 282; 190; 177; 137.

*7-*(*3,5-Dichlorophenylamine*)*-5-methyl-2-*(*trifluoromethyl*)[*1,2,4*]*triazolo*[*1,5-a*]*pyrimidine* (**13**). Yield: 85%; m.p. 207–208 °C. IR (KBr, cm^−1^): 3,319 (NH); 3,084; 3,062; 1,624 (C=N); 1,560; 1,510; 1,433; 1,371; 1,319; 1,286 (C=N); 1,193; 1,174; 1,143; 806; 779 (CF_3_). ^1^H-NMR (CD_3_OD, δ): 2.54 (s; 3H; CH_3_); 6.61 (s; 1H; H-6); 7.41 (t; *J* = 0.9 Hz; 1H; H-4'); 7.48 (d; *J* = 1.2 Hz; 2H; H-2', H-6'). ^13^C-NMR (CD_3_OD, δ): 25.38; 93.03; 121.11 (q; *J* = 215 Hz; C-9); 124.54; 128.02; 137.37; 140.50; 148.11; 156.94 (q; *J* = 31.3 Hz; C-2); 157.41; 169.11. ^19^F-NMR (CD_3_OD, δ): −67.23 (s; 3F). GC/MS *m/z* (%): 361; 362; 363; 346; 292; 190 (100); 177; 145.

*7-*(*3,5-Dimethoxyphenylamine*)*-2,5-dimethyl*[*1,2,4*]*triazolo*[*1,5-a*]*pyrimidine* (**14**). Yield: 90%; m.p. 112–114 °C. IR (KBr, cm^−^^1^): 3,518 (NH); 1,629 (C=N); 1,585; 1,519; 1,483; 1,388; 1,357; 1,311; 1,284; 1,203; 1,155; 931; 817. ^1^H-NMR (CD_3_OD, δ in ppm): 2.45 (s, 3H, CH_3_); 2.52 (s, 3H, CH_3_); 3.80 (s, 6H, OCH_3_); 6.42 (s, 1H, H-6); 6.44 (dd, *J* = 1.6 Hz, 1H, H-4'); 6.56 (d, *J* = 2.0 Hz; 2H; H-2', H-6'). ^13^C-NMR (CD_3_OD, δ in ppm): 14.78; 24.96; 56.17; 91.03; 99.49; 103.81; 139.51; 147.41; 157.23; 163.28; 165.29; 166.38. GC/MS *m/z* (%): 299 (100); 298; 284; 269; 136; 123.

*7-*(*3,5-Dichlorophenylamine*)*-2,5-dimethyl*[*1,2,4*]*triazolo*[*1,5-a*]*pyrimidine* (**15**). Yield: 85%; m.p. 128–130 °C. IR (KBr, cm^−^^1^): 3,560 (NH); 3,138 (CH-aromatic); 1,612 (C=N); 1,591; 1,564; 1,525; 1,454; 1,371; 1,361; 1,321; 1,114; 935; 835; 804. ^1^H-NMR (CD_3_OD, δ): 2.49 (s, 3H, CH_3_); 2.52 (s, 3H, CH_3_); 6.48 (s, 1H, H-6); 7.39 (dd, *J* = 1.2 Hz, 1H, H-4'); 7.45 (d, *J* = 1.2 Hz; 2H; H-2', H-6'). ^13^C-NMR (CD_3_OD, δ): 14.80; 25.01; 91.24; 123.86; 127.37; 123.18; 137.37; 140.77; 146.75; 157.22; 165.48; 166.87. GC/MS *m/z* (%): 307; 309; 272; 145; 136 (100); 123; 109.

*2,5-Dimethyl-7-*(*4-*(*trifluoromethyl*)*phenylamine*)*-*[*1,2,4*]*triazolo*[*1,5-a*]*pyrimidine* (**16**). Yield: 80%; m.p. 191–193 °C. IR (KBr, cm^−1^): 3,211 (NH); 1,328; 1,604 (C=N); 1,564; 844. ^1^H-NMR (CD_3_OD, δ): 2.47 (s, 3H, CH_3_); 2.52 (s, 3H, CH_3_); 6.54 (s, 1H, H-6); 7.77 (d, *J* = 8.0 Hz, 2H, H-2', H-6'); 7.63 (d, *J* = 8.0 Hz; 2H; H-3', H-5'). ^13^C-NMR (CD_3_OD, δ): 14.76; 24.98; 91.19; 125.12; 125.63 (q; *J* = 215 Hz; CF_3_); 128.09; 129.05 (q; *J* = 215 Hz; C4'); 141.96; 146.70; 157.30; 165.51; 166.85. ^19^F-NMR (CD_3_OD, δ): −63.79 (s, 3F). GC/MS *m/z* (%): 307 (100); 145; 136; 123. 

*7-*(*3,5-Dichlorophenylamine*)*-2,5-bis*(*trifluoromethyl*)[*1,2,4*]*triazolo*[*1,5-a*]*pyrimidine* (**17**). Yield: 50%; m.p 248–249 °C. IR (KBr, cm^−1^): 3,624 (NH); 3,313; 3,120; 1,658 (C=N); 1,622; 1,591; 1,510; 1,454; 1,394; 1,323; 1,301; 1,217; 1,163; 1,139; 1,051; 968; 858; 773 (CF_3_). ^1^H-NMR (CD_3_OD, δ): 7.17 (t; *J* = 1.2 Hz; 1H; H-4’); 7.22 (s; 1H; H-6); 7.90 (d; *J* = 1.2 Hz; 2H; H-2', H-6'). ^13^C-NMR (CD_3_OD, δ): 106.17; 119.68; 120.07 (q; *J* = 217.5 Hz; C-10); 120.68 (q; *J* = 214.7 Hz; C-9); 125.10; 136.39 (q; *J* = 31 Hz; C-5); 136.49; 142.02; 157.13 (q; *J* = 31 Hz; C-2); 157.91; 158.37. ^19^F-NMR (CD_3_OD, δ): −67.82 (s; 3F); −70.08 (s; 3F); GC/MS *m/z* (%): 414 (100); 415; 417; 396; 380; 345; 145.

*7-Cyclohexylamine-5-methyl-2-*(*trifluoromethyl*)[*1,2,4*]*triazolo*[*1,5-a*]*pyrimidine* (**18**). Yield: 75%; m.p 128–130 °C. IR (KBr, cm^−1^): 3,344 (NH); 3,012; 2,941; 2,860; 2,798 (C–H); 1,624 (C=N); 1,589; 1,516; 1,452; 1,427; 1,367; 1,321; 1,303; 1,286; 1,203; 1,172, 1,155; 796 (CF_3_). ^1^H-NMR (CD_3_OD, δ): 1.24–3.67 (m; 11H, CH_2_ and CH); 6.47 (s; 1H; H-6). ^13^C-NMR (CD_3_OD, δ): 25.0; 26.14; 26.39; 33.22; 53.37; 90.83; 120.92 (q; *J* = 214.9 Hz; C-9); 148.68; 156.34 (q; *J* = 31.1 Hz; C-2); 157.17; 167.93. ^19^F-NMR (CD_3_OD, δ): −67.28 (s; 3F; F-9); GC/MS *m/z* (%) 299; 280; 270; 230; 217 (100); 190; 177.

*4-*(*5-Methyl-7-*(*4-morpholinyl*)*-2-*(*trifluoromethyl*)[*1,2,4*]*triazolo*[*1,5-a*]*pyrimidine* (**19**). Yield: 70%; m.p. 215–216 °C. IR (KBr, cm^−1^): 3,051; 2,972; 2,912; 2,960; 2,870 (C–H); 1618 (C=N); 1,564; 1,442; 1,371; 1,323; 1,296; 1,274; 1,213; 1,197; 1,180; 1,120; 773; (CF_3_). ^1^H-NMR (CD_3_OD, δ): 3.91 (m, 8H, CH_2_); 2.58 (s, 3H, CH_3_); 6.63 (s, 1H, H-6). ^13^C-NMR (CD_3_OD, δ): 25.04; 49.94; 67.35; 97.35; 120.95 (q, *J* = 268.8 Hz; C-9); 156.04 (q, *J* = 39.1 Hz; C-2); 152.01; 158.75; 168.57. ^19^F-NMR (CD_3_OD, δ): −67.46 (s, 3F). GC/MS *m/z* (%): 287 (100); 288; 268; 256; 244; 230; 218; 202; 177.

*5-Methyl-7-*(*4-methylpiperazin-1-yl*)*-2-*(*trifluoromethyl*)[*1,2,4*]*triazolo*[*1,5-a*]*pyrimidine* (**20**). Yield: 90%; m.p. 158–160 °C. IR (KBr, cm^−1^): 3,047; 2,976; 2,947; 2,910; 2,850; 2,800 (C-N); 1,618 (C=N); 1,554; 1,512; 1,450; 1,431; 1,371; 1,323; 1,294; 1,213; 1,190; 1,141; 777 (CF_3_). ^1^H-NMR (CD_3_OD, δ): 2.38 (s, 3H, CH_3_); 2.56 (s, 3H, N-CH_3_); 2.68 (m, 4H); 3.94 (m, 4H); 6.63 (s, 1H, H-6). ^13^C-NMR (CD_3_OD, δ): 25.03; 46.13; 49.19; 55.34; 97.56; 120.95 (q, *J* = 268.5 Hz, C-9); 151.86; 156.00 (q, *J* = 39.1 Hz, C-2); 158.72; 168.44. ^19^F-NMR (CD_3_OD, δ): −67.39 (s, 3F). GC/MS *m/z* (%): 300; 285; 231; 70 (100).

*5-Methyl-7-*(*pyrrolidin-1-yl*)*-2-*(*trifluoromethyl*)[*1,2,4*]*triazolo*[*1,5-a*]*pyrimidine* (**21**). Yield: 90%; m.p. 234–236 °C. IR (KBr, cm^−1^): 2,987; 2,949; 2,877 (C-N); 1,629 (C = N); 1,571; 1,508; 1,444; 1,371; 1,355; 1,309; 1,261; 1,211; 1,192; 1,138; 771 (CF_3_). ^1^H-NMR (CD_3_OD, δ): 2.07–2.10 (m, 8H); 2.55 (s, 3H, CH_3_); 6.19 (s, 1H, H-6). ^13^C-NMR (CD_3_OD, δ): 24.71; 26.47; 52.36; 121.04 (q, *J* = 268.4 Hz, C-9); 149.40; 155.84 (q, *J* = 38.6 Hz, C-2); 159.00; 167.12. ^19^F-NMR (CD_3_OD, δ): −67.79 (s, 3F). GC/MS *m/z* (%): 271; 252; 202 (100).

### 3.4. General Procedure for Preparing 5-Methyl-7-substituted[1,2,4]triazolo[1,5-a]pyrimidines *22–27*

A mixture of 7-chloro-5-methyl-2-(trifluoromethyl)-[1,2,4]triazolo[1,5-*a*]pyrimidine (**3c**) and the appropriate azole or amine (1 equivalent) in DMF (3 mL) was stirred at 120 °C for 12 h. The organic solvent was then removed under reduced pressure. The solid was collected and recrystallized from EtOH/H_2_O to give **22**–**27** a yield of 50–72%.

*5-Methyl-7-*(*pyridin-2-ylamine*)*-2-*(*trifluoromethyl*)[*1,2,4*]*triazolo*[*1,5-a*]*pyrimidine* (**22**). Yield: 50%; m.p 174–177 °C. IR (KBr, cm^−1^): 3,643 (NH); 3,161 (CH-aromatic); 1,631 (C=N); 1,136; 775 (CF_3_). ^1^H-NMR (CD_3_OD, δ): 2.64 (s; 3H; CH_3_); 7.81 (s; 1H; H-6); 8.43–7.16 (m; 4H; H-3'-H-6'); 8.22 (s; NH). ^13^C-NMR (CD_3_OD, δ): 25.64; 97.34; 115.97; 120.91; 121.01 (q; *J* = 215.0 Hz; C-9); 139.86; 145.21; 148.87; 153.40; 156.39 (q; *J* = 31.0 Hz; C-2); 156.81; 169.33. ^19^F-NMR (CD_3_OD, δ): −67.47 (s; 3F; F-9).

*5-Methyl-2-*(*trifluoromethyl*)*-7-*(*5-*(*trifluoromethyl*)*-3-amine-4H-1,2,4-triazolyl*)[*1,2,4*]*triazolo*[*1,5-a*]*pyrimidine* (**23**). Yield: 60%; m.p. 217–219 °C. IR (KBr, cm^−1^): 3,398; 3,334 (NH); 3,111 (CH-aromatic); 1,643; 1,633 (C=N); 1,190; 758; 750 (CF_3_). ^1^H-NMR (CD_3_OD, δ): 2.83 (s, 3H, CH_3_); 7.73 (s, 1H, H-6). ^13^C-NMR (CD_3_OD, δ): 25.6; 93.7; 121.01 (q, *J* = 268.5 Hz, C-9); 125.59 (q, *J* = 269 Hz, C-18); 125.75; 128.14; 129.61 (q, *J* = 33 Hz, C-15); 140.1; 147.97; 156.85 (q, *J* = 39 Hz, C-2); 158.0; 159.7. ^19^F-NMR (CD_3_OD, δ): −67.20 (s, 3F); −68.53 (s, 3F). GC/MS *m/z* (%): 352 (100); 333; 310; 283.

*5-Methyl-7-*(*1H-pyrazol-1-yl*)*-2-*(*trifluoromethyl*)[1,2,4]*triazolo*[*1,5-a*]*pyrimidine* (**24**). Yield: 70%; m.p. 221–223 °C. IV (KBr, cm^−1^): 3,128 (NH); 1,631; 1,566; 1,195; 1,153; 783 (CF_3_). ^1^H-NMR (CD_3_OD): 2.79 (s, 3H, CH_3_), 7.93 (s, 1H, H-6); 9.38 (d, *J* = 2.8 Hz, H-3'); 8.03 (d, *J* = 1.6 Hz, H-5'); 6.78 (dd, *J* = 2.8 Hz, 1.6 Hz, H-4'). ^13^C-NMR (CD_3_OD, δ): 25.58; 30.81; 102.79; 111.56; 120.78 (q, *J* = 270 Hz, C-9); 134.82; 143.49; 146.32; 157.70 (q, *J* = 40 Hz, C-2); 170.64. ^19^F-NMR (CD_3_OD, δ): −67.38 (s, 3F). GC/MS *m/z* (%): 268 (100); 119.

*7-*(*1H-Imidazol-1-yl*)*-5-methyl-2-*(*trifluoromethyl*)[*1,2,4*]*triazolo*[*1,5-a*]*pyrimidine* (**25**). Yield: 72%; m.p. 157–159 °C. IV (KBr, cm^−^^1^): 3,159 (NH); 3,047; 1,631(C=N); 1,192; 783 (CF_3_). ^1^H-NMR (CD_3_OD, δ): 2.81 (s, 3H, CH_3_); 7.74 (s, 1H, H-6); 7.33 (s, 1H, H-5'); 8.14 (s, 1H, H-4'); 8.87 (s, 1H, H-2'). ^13^C-NMR (CD_3_OD, δ): 25.58; 104.92;. 120.73 (q, *J* = 270 Hz, C-9); 120.91; 131.16; 139.70; 141.39; 157.71 (q, *J* = 40 Hz, C-2); 171.21. ^19^F-NMR (CD_3_OD, δ): −67.37 (s, 3F); GC/MS *m/z* (%):268 (100); 241; 214; 146.

*5-Methyl-7-*(*1H-1,2,4-triazol-1-yl*)*-2-*(*trifluoromethyl*)[*1,2,4*]*triazolo*[*1,5-a*]*pyrimidine* (**26**). Yield: 65%; m.p. 173–175 °C. IV (KBr, cm^−^^1^): 3,076; 1,639 (C=N); 1,566; 1,193; 777 (CF_3_). ^1^H-NMR (CD_3_OD, δ): 2.84 (s, 3H, CH_3_), 7.99 (s, 1H, H-6); 8.43 (s, 1H, H-5'); 9.98 (s, 1H, H-2'). ^13^C-NMR (CD_3_OD, δ): 25.75; 103.95; 120.69 (q, *J* = 214 Hz, C-9); 141.01; 148.31; 154.58; 157.85 (q, *J* = 32 Hz, C-2); 171.41. ^19^F-NMR (CD_3_OD, δ): −67.59 (s, 3F); GC/MS *m/z* (%): 269 (100); 250; 242; 92.

*5-Methyl-7-*(*1H-pyrrol-1-yl*)*-2-*(*trifluoromethyl*)[*1,2,4*]*triazolo*[*1,5-a*]*pyrimidine* (**27**). Yield: 60%; m.p. 198–200 °C. IV (KBr, cm^−^^1^): 3,375; 1,620 (C=N); 1,192; 744 (CF_3_). ^1^H-NMR (CD_3_OD, δ): 2.69 (s, 3H, CH_3_), 7.57 (s, 1H, H-6); 7.67–6.46 (m, 4H). ^13^C-NMR (CD_3_OD, δ): 25.12; 106.29; 112.82; 119.50; 120.91 (q, *J* = 269 Hz, C-9); 127.98; 141.05; 157.29 (q, *J* = 50 Hz, C-2); 168.30; ^19^F-NMR (CD_3_OD, δ): −67.20 (s, 3F). GC/MS *m/z* (%): 267 (100).

### 3.5. General Procedure for Preparing 5-Methyl-7-N'-(N,N-diethylpentane-1,4-diamine)[1,2,4]triazolo [1,5-a]pyrimidines *28, 29*

A mixture of a 7-chloro[1,2,4]triazolo[1,5-*a*]pyrimidine derivative **3a**,**c** (1 mmol) and the appropriate amine (1.5 mmol) in ethanol (10 mL) was stirred at room temperature for 43 h. Excess solvent was removed under reduced pressure, giving the respective derivative **28** and **29** a yield of 80–83%, as pale yellow oils.

*5-Methyl-7-N'-*(*N,N-diethylpentane-1,4-diamine*)[*1,2,4*]*triazolo*[*1,5-a*]*pyrimidine* (**28**). Yield: 80%. IV (neat, cm^−1^): 3,420 (NH); 1,651; 1,601 (C=N); 1,219; 1,149; 992. ^1^H-NMR (acetone-*d_6_*, δ): 1.03 (t; 6H; *J* = 7 Hz, CH_2_-CH_3_); 1.23 (d; 3H; *J* = 7.5 Hz, CH_3_); 1.35 (m; 4H, CH_2_-CH_2_); 2.01 (s; 3H, CH_3_); 2.56–2.45 (m; 7H, CH_2_-CH_3_; CH_2_ and CH); 4,12 (sl; 1H, NH); 6,38 (s; 1H, H-6); 8,26 (s; 1H, H-2). ^13^C-NMR (acetone-*d_6_*, δ): 13.3; 16.20; 19.9; 22.9; 24.0; 30.6; 46.7; 53.8; 101.1; 140.9; 154.2; 158.5; 162.7. GC/MS *m/z* (%): 290; 275; 230; 218; 86 (100).

*5-Methyl-7-N'-*(*N,N-diethylpentane-1,4-diamine*)*-2-*(*trifluoromethyl*)[*1,2,4*]*triazolo*[*1,5-a*]*pyrimidine* (**29**). Yield: 83%. IV (neat, cm^−1^): 3,434 (NH); 1,650; 1,604 (C=N); 1,218; 1,151; 995; 772 e 747 (CF_3_). ^1^H-NMR (400 MHz, CD_3_OD, δ): 1.06 (t; 6H; *J* = 8 Hz, CH_2_-CH_3_); 1.18 (d; 3H; *J* = 6 Hz, CH_3_); 1.70–1.45 (m; 4H, CH_2_-CH_2_); 2.56 (s; 3H, CH_3_); 2.80–2.45 (m; 7H, CH_2_-CH_3_; CH_2_ and CH); 4.15 (sl; 1H, NH); 6.12 (s; 1H, H-6). ^13^C-NMR (100 MHz, CD_3_OD, δ): 9.7; 16.0; 19.3; 21.8; 24.2; 33.5; 45.9; 51.9; 101.1; 118.9 (q; *J* = 269 Hz; C-9); 144.9; 152.2 (q; *J* = 14.5 Hz; C-2); 158.5; 163.7. ^19^F-NMR (376 MHz, CD_3_OD, δ): −66.43 (s, 3F); GC/MS *m/z* (%):358; 86 (100); 69; 58.

## 4. Conclusions

One important strategy in drug design is the chemical modification of available drugs to develop novel, biologically active compounds. This approach seeks to improve the “druggability” of analogues, thus reducing the chance of causing parasite resistance [43]. We have synthesized 26 new derivatives of the [1,2,4]triazolo[1,5-*a*]pyrimidine system, with different substituents at the 2-, 5- and 7-positions of that ring system; these compounds exhibited a range of anti-*P. falciparum* activities. The data suggest that these compounds can be used as potential agents against malaria.

The results show that compounds containing an arylamine substituent in the 7-position of [1,2,4]triazolo[1,5-*a*]pyrimidine exhibit anti-plasmodial activity against the W2 chloroquine-resistant *P. falciparum* clone, with IC_50_ values of 0.023 to 20 µM. This trend is exemplified by compounds **5** (2-naphthyl), **8** (3,4-diCl), **11** (4-OCH_3_), and **16** (4-CF_3_). In compound **5** the naphythylamine substituent at the 7-position has an important contribution to anti-*Plasmodium falciparum* activity, when compared with compounds **8** and **11**, which contain arylamine groups. However, compound **16** having 4-CF_3_-phenylamine as a substituent at 7-position was more important than CF_3_ group at 2-position.

None of these compounds were toxic to HepG2 cells. The substituent groups at the 7-position of the [1,2,4]triazolo[1,5-*a*]pyrimidine ring were found to play an important role in the anti-*Plasmodium* activity. The trifluoromethyl group as a substituent at the 2-position of the [1,2,4]triazolo[1,5-*a*]pyrimidine ring contributed to increased anti-plasmodial activity in several compounds (**5**, **8**, **11**). 

Docking simulations of the synthesized compounds with *Pf*DHODH are in accordance with the crystallographic investigation published elsewhere [32], which suggests that the presence of “molecular anchors” formed by specific hydrogen bonds between the ligands and the enzyme should be considered carefully for the design of potential new lead compounds. The residues involved in these hydrogen bonds are H185 and R265. Moreover, additional hydrogen bonds between nearly all of the compounds and a water molecule (W15) should also be considered for the stabilization of the ligand-enzyme interaction. The presence of a CF_3_ group can facilitate such hydrogen bonds.
